# Computer-aided Design and Manufacturing Crown on Primary Molars: An Innovative Case Report

**DOI:** 10.5005/jp-journals-10005-1591

**Published:** 2019

**Authors:** Petros Mourouzis, Aristidis Arhakis, Kosmas Tolidis

**Affiliations:** 1,3 Department of Operative Dentistry, Aristotle University of Thessaloniki, Thessaloniki, Greece; 2 Department of Paediatric Dentistry, Aristotle University of Thessaloniki, Thessaloniki, Greece

**Keywords:** CAD/CAM, Hybrid ceramic crown, Primary molars

## Abstract

**Aim:**

Crowns are applicable on primary teeth with extensive caries, cervical decalcification, developmental defects, interproximal caries extending beyond line angles, and following pulpotomy or pulpectomy.

**Background:**

Until now, prefabricated crowns, i.e., stainless-steel crowns (SSCs), pre-veneered SSC, and all ceramic/porcelain/zirconia crowns have been available in a range of sizes to match the first and the second primary molar teeth.

**Case description:**

This case report illustrates a clinical use of computer-aided design and manufacturing technology (CAD/CAM) for the fabrication of a crown for a decayed primary molar using a study model as a reference. The material used was a hybrid ceramic CAD/CAM block.

**Conclusion:**

This approach has the advantage of the customization of the abutment tooth in contrast to the previously mentioned prefabricated options.

**How to cite this article:**

Mourouzis P, Arhakis A, *et al.* Computer-aided Design and Manufacturing Crown on Primary Molars: An Innovative Case Report. Int J Clin Pediatr Dent 2019;12(1):76–79.

## INTRODUCTION

The use of stainless steel crowns (SSCs) for primary molars in pediatric dentistry is a common practice in the management of heavily decayed and deformed primary teeth.^1,2^ The crowns provide a solution for a restoration with the highest success rate, without causing secondary caries and are cost effective.^3^ The metallic color appearance of SSCs, the possible damage to gingival tissues, and the possibility of cytotoxic and allergenic phenomena due to the release of nickel and chromium ions into the saliva may promote biocompatibility issues.^4^ Prefabricated zirconia crowns for primary teeth were introduced in 2010 as an alternative and more aesthetic option to SSCs.^5^ Manufacturers offer a significant range of zirconia crown sizes, along with a specific preparation and cementation protocol.^6^ Unlike SSC, they cannot be modified in any way, they are incapable of withstanding flexure, and may fracture on cementation.^5^

Even though prefabricated zirconia crowns provide acceptable tooth color, there is a limited selection of restoration shades and contours, while some of the marketed brands require over 2 mm of tooth reduction.^7^ Moreover, prefabricated zirconia crowns require feather-edged subgingival preparation, thus, potentially extending the operatory time due to the gingival injuries that may occur.^7^

Computer-aided design and manufacturing (CAD/CAM) technology have made enormous improvements since its introduction by Dr Francois Duret^8^ and Dr Werner Mormann.^9^ Nowadays, this technology is available directly in dental clinics and it is capable, *via* its software, of fabricating (customized) full ceramic crowns, inlays, onlays, and veneers for permanent dentition at one appointment. The materials that are used in CAD/CAM include ceramic, resin ceramic, hybrid ceramic, and zirconia blocks. The superior mechanical properties of these materials support the use of CAD/CAM as a trustworthy method for dental patients because it results in a high survival rate of the restorations with a low rate of restoration fracture and long-term clinical survivability.^10^

The purpose of this paper is to illustrate a case report of the fabrication of a CAD/CAM crown for a decayed primary maxillary molar in an 8-year-old boy.

## CASE PRESENTATION

### Clinical Examination

**Fig. 1:**
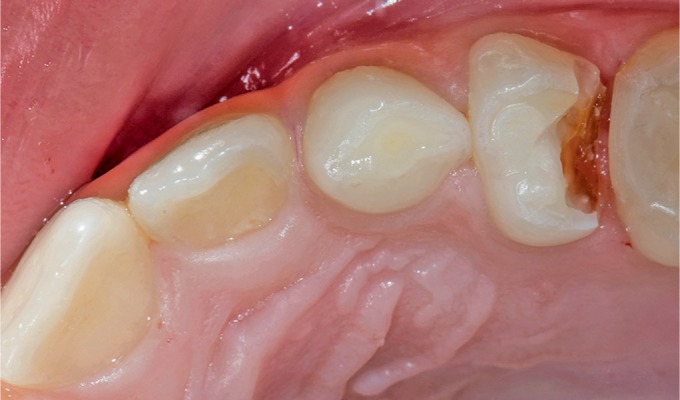
Intraoral view of the carious upper left maxillary first primary molar

**Fig. 2:**
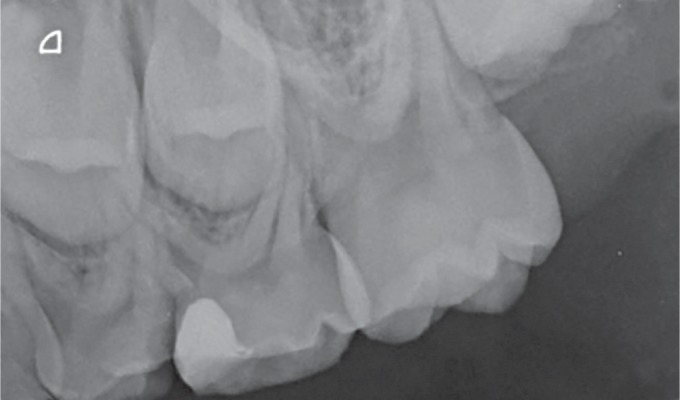
X-ray evaluation of the upper left maxillary primary molar

**Fig. 3:**
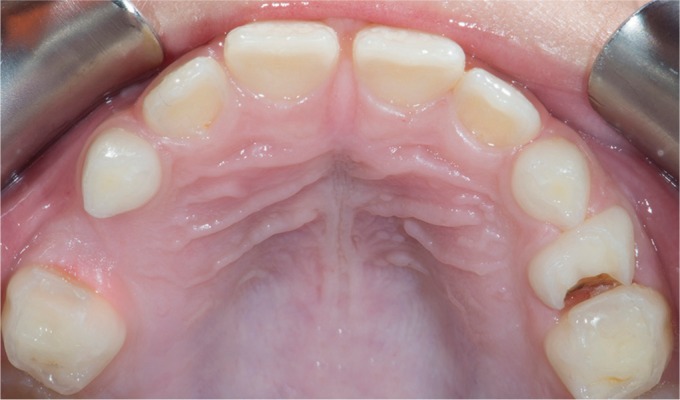
Occlusal view of the previously extracted primary tooth #54

**Fig. 4:**
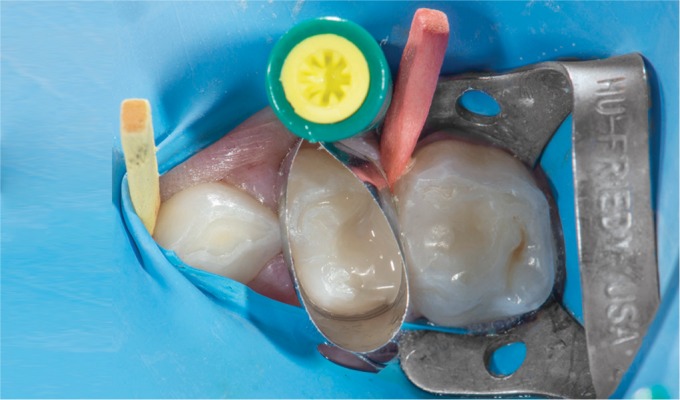
Intraoral picture of the tooth after the placement of the filling material

**Fig. 5:**
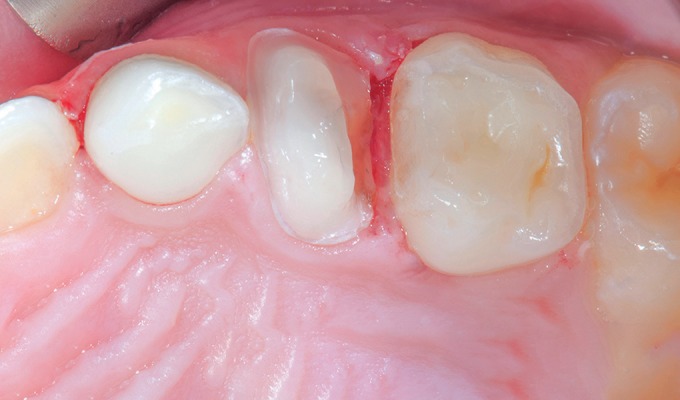
The core built-up ready for the scanning procedure

An 8-year-old female patient presented at our clinic complaining of pain due to food impaction in the upper left maxillary primary molar area. A medical history was taken, followed by a clinical and radiographic examination which revealed deep dentinal caries on the tooth in question tooth without any interradicular lesion (Figs 1 and 2). Furthermore, on the other quadrant, the complementary molar had been previously extracted as a result of pathologic bone resorption accompanied by corresponding external root resorption due to caries (Fig. 3). Likewise, due to patient's age and the extensive, multisurface restoration needed on the upper left maxillary primary molar, the treatment plan suggested a crown for that tooth.^11^ Moreover, patient's parents refused the SSC treatment option due to aesthetic reasons, while they request to avoid the extraction of the tooth due to negative experience from the extraction of the upper right maxillary primary molar. Topical infiltrative anesthesia was administered (2% lidocaine with 1:100,000 adrenaline), the tooth was isolated with a rubber dam, and the caries were removed with a high-speed handpiece and a carbide bur (no. 330). Selective decay removal was performed with low-speed round burs until the remaining dentin was rigid and free of decay, after the decay removal, the gingival wall was 1.0 mm under the cementoenamel junction. For this reason, a proximal box elevation was performed so that the margin in the gingival wall was at the gingival level (Fig. 4). The resin used for core buildup was Tetric Evoceream (Ivoclar Vivadent, Schaan, Liechtenstein). The tooth was then prepared with the diamond bur round end taper no. 8881-314-014 for an axial reduction of 0.8–1.0 mm, followed by a chamfer margin circumferentially and occlusal reduction of 1.0–1.5 mm with a round wheel no. 909—(Κomet, Brasseler, Lemgo, Germany) according to the recommended preparation guidelines of the manufacture of the hybrid ceramic block (Fig. 5).

### Intraoral Scanning and Milling

**Fig. 6:**
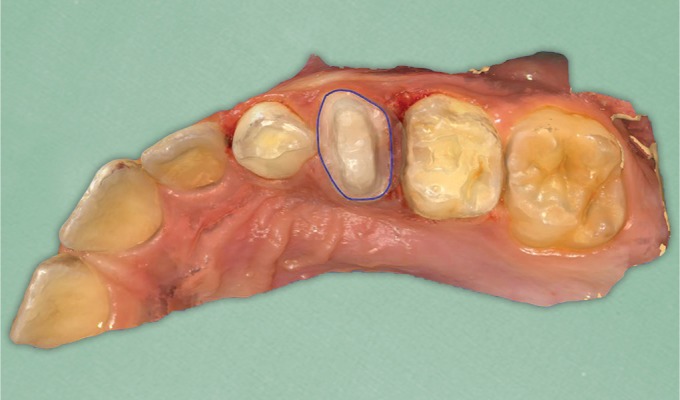
Digital image from the CEREC SW 4.6. Design of the margin of the restoration

**Fig. 7:**
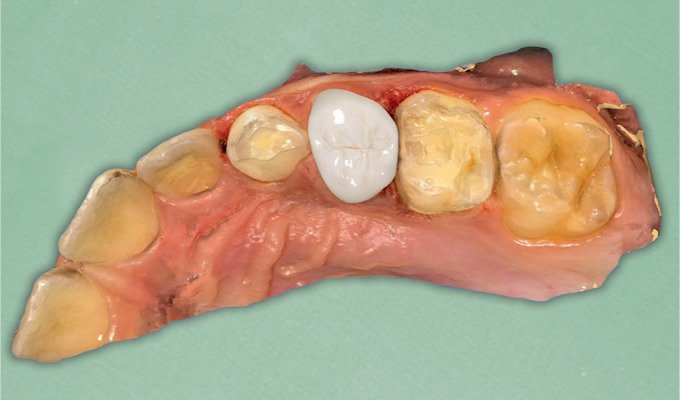
Digital image from the CEREC SW 4.6. The digital restoration seated

**Fig. 8:**
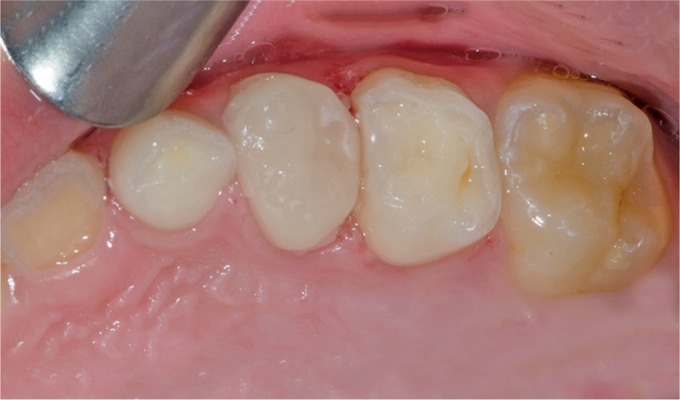
Final occlusal view of the restored tooth

**Fig. 9:**
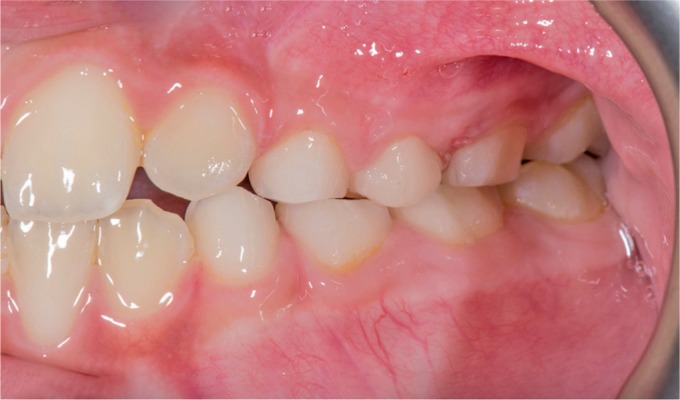
Twelve months follow-up examination. Buccal view of the final restoration

To allow a more effective scan with the relative isolation of the treatment field, Optragate (Ivoclar, Vivadent, Liechtenstein) was applied to ensure the lips and cheeks were evenly retracted. Quarters arch scans were made using a powder-free intraoral scanning device (Cerec AC, Omnicam, Sirona Dental Systems GmbH, Bensheim, Germany) (Video 1). The design mode of the restoration was completed using the Biogeneric Copy (Cerec SW 4.6) and enabling the program to copy a primary tooth no. 64 from a study children's model (Kavo Dental, Charlotte, USA). The software optical images included, as stated: the “upper jaw,” the “lower jaw,” the “buccal,” and the “biocopy upper.” In the next step, the software automatically aligns the upper and lower jaws and articulates the models in the maximum intercuspal position. The gingival margins were defined automatically and manually designed using the “draw margin” tool (Fig. 6). Cerec SW 4.6 automatically calculates the insertion axis and also provides tools to adjust the restoration design, including the occlusal and interproximal occlusal contact points. In the milling preview, the restoration was placed in a hybrid ceramic block (Vita Enamic, Vita Zahnfabrik, H. Rauter GmbH & Co. KG, Germany) with a shade of 2M2-HT and EM-10 size (LOT 56802, REF20170404), that was automatically determined by the software with the shade analysis tool. The milling of the block was completed in the “Standard” mode and milled with the CEREC MC X milling unit and diamond burs (step bur 12S, cylinder pointed bur 10) (Fig. 7). After the completion of the milling procedure, the crown was hand polished according to the specifications from the manufacturer. The restoration was cleaned with alcohol and dried with oil- and water-free air. The inner surface of the crown was sandblasted with Al_2_O_3_ at two bar pressure followed by etching with 5% hydrofluoric acid for 60 seconds and then placed in an ultrasonic bath for 5 minutes. The crown was then cemented with the self-adhesive resin cement (Solocem, Coltene, Whaledent, Altstatten, Switzerland) according to the instructions from the manufacturer and polymerized with a Bluephase LED device at 1.200 mW/cm^2^ (Ivoclar Vivadent, Schaan, Liechtenstein). The resin cement was set and excesses were removed from the interproximal space with dental floss, the occlusion was checked, and instructions for oral hygiene were given. The editing time of the restoration had a duration of 2 minutes, the milling time took 9 minutes, while the total chairside time was 50 minutes. Initial and final intraoral pictures are presented in Figures 8 and 9.

## DISCUSSION

This case report describes a single-visit fabrication of a hybrid ceramic crown on a primary molar. To limit chairside time and promote the quality of care given, CAD/CAM technology could be beneficially used in cases when a crown on primary tooth is needed and the option of extraction is rejected by the parents of the patient, while the cost of extraction and placement of a space retainer is equivalent to the fabrication of a CAD/CAM crown. SSCs in young patients are the standardized treatment option for heavily decayed primary teeth.^11^ They are used in cases where a primary molar has multisurface lesions, is anticipated to exfoliate in the far future, or has been treated with pulpotomy/pulpectomy.^12^ The biggest disadvantage of SSCs is the aesthetically unattractive result.^13^ To overcome the poor aesthetics, new materials were developed, such as the open-faced crowns or pre-veneered SSCs. However, those materials have displayed major drawbacks such as chipping of the buccal facade due to higher chewing forces, poor gingival health, or the exposure of dental margins.^14^ Another deficiency of SSCs is that there is a potential for nickel and chromium ion release into the intraoral environment or into the tooth root tissue and this can possibly result in an allergic reaction or may be cytotoxic.^4,15,16^

Vita Enamic is a hybrid ceramic material containing a porous sintered ceramic network filled with plastic and consisting of two interlocking networks: a ceramic and a polymer network, termed a double-network hybrid.^17^

The monomers that are incorporated are urethane dimethacrylate (UDMA) and triethylene glycol dimethacrylate (TEGDMA) (14% wt–25% v/v) and characterized by the acronym P.I.C.N. which stands for Polymer-Infiltrated-Ceramic-Network.^18^ Vita Enamic has an elastic modulus of 30–32 GPa and resembles dental hard tissues, enabling it to mimic the biomechanical properties of natural teeth.^19^ Moreover, Vita Enamic has the advantage of preserving the antagonist teeth from abrasion and performing better at heavy occlusal stresses because it has the ability to resist crack initiation and progression.^20^ CAD/CAM technology is an innovative method of producing indirect restorations in primary teeth. With the help of the design software, the clinician can produce ideal occlusal and proximal contact points and a better marginal fit at the gingival wall.^21^ Moreover, it involves a shorter clinical working time, less wear of the opposing dentition, and the choice of more biocompatible materials.^22^ To the best of our knowledge, this is the first case report of a CAD/CAM crown on a first primary molar in the literature. However, there are limited cases of restorations on primary teeth with CAD/CAM technology that are associated with the fabrication of inlays or onlays for a primary decayed tooth or the fabrication of an endocrown.^23^

In this case report, the restoration was performed with a newly introduced material (Vita Enamic). The novelty of the case report is that the CAD/CAM software copied a primary tooth from a study children's model so that a plastic primary tooth #64 could serve as a prototype. This was performed because CEREC SW 4.6 does not contain a primary teeth database. Finally, the restoration had a high aesthetical outcome, while the hybrid ceramic material facilitates maximum adhesion when a self-adhesive dual-cure resin cement is used by virtue of the polymer network. It is not a sensitive technique, with comparable chairside treatment time with SSC and zirconia crowns.^24^

To conclude, the tooth is vital and over a 12-month follow-up period, no pulpal, periodontical, or periradicular pathology was detected. The restoration performance is excellent exhibiting no chipping, no discoloration, and displays an excellent marginal fit. The patient was placed under a prevention treatment plan of high-risk caries.

## CONCLUSION/RECOMMENDATION

Although there is no published literature for CAD/CAM restorations for primary molars, there are dental clinics that use CAD/CAM for the fabrication of restorations to permanent dentition and occasionally to primary teeth. This case report illustrates an alternative and reliable treatment option for primary molars with extensive caries and possesses the advantage of limiting chairside time and full customization of the crown restoration together with a high level of aesthetic outcome.

## CLINICAL SIGNIFICANCE

This type of approach could be considered as an alternative option for primary molar with extensive caries, having the advantage of limiting chairside time and a customized procedure.
